# Parents’ Experiences After Neonatal Intensive Care Unit Discharge: A Qualitative Descriptive Study

**DOI:** 10.3390/nursrep16100373

**Published:** 2026-10-20

**Authors:** Adelaide D’Errico, Gianluca Azzellino, Biagio Nicolosi, Emanuele Buccione, Giorgia Grandville, Camilla Elena Magi, Laura Rasero, Stefano Bambi, Roberta Previdera

**Affiliations:** 1 Santobono-Pausilipon Children’s Hospital, 80122 Naples, Italy; giograndville@gmail.com (G.G.); r.previdera@santobonopausilipon.it (R.P.); 2 Department of Life, Health and Environmental Sciences, University of L’Aquila, 67100 L’Aquila, Italy; gianluca.azzellino@graduate.univaq.it; 3 Adriatic District Area, Local Health Authority 4 of Teramo, 64100 Teramo, Italy; 4 Health Professions Department, Meyer Children’s Hospital IRCCS, 50139 Florence, Italy; biagio.nicolosi@meyer.it; 5 Pediatric Intensive Care Unit, Local Health Authority 3 of Pescara, 65124 Pescara, Italy; 6 Department of Health Sciences, University of Florence, 50139 Florence, Italy; camillaelena.magi@unifi.it (C.E.M.); l.rasero@unifi.it (L.R.); stefano.bambi@unifi.it (S.B.)

**Keywords:** neonatal intensive care, parents’ experiences, transitional care, qualitative description, thematic analysis, continuity of care, post-discharge support

## Abstract

**Background/Objectives:** Transition from the Neonatal Intensive Care Unit (NICU) to home involves emotional adjustment and increasing parental responsibility for infant care. This study explored parents’ experiences of this transition, focusing on emotional experiences, perceived continuity of care, and support needs. **Methods:** A qualitative descriptive study included 38 parents representing 34 preterm or medically complex infants discharged from a single NICU in an Italian tertiary children’s hospital. Individual semi-structured interviews were conducted within 12 months of discharge, audio-recorded, and transcribed in Italian. Predominantly inductive thematic analysis examined shared meanings and variations across parents’ retrospective interview accounts. Recruitment was time-limited; neither saturation nor information power was formally assessed. **Results:** Four themes were developed: returning home with joy as the focus of fear changes; being with the infant and learning to provide care; remaining in contact without always feeling accompanied; and receiving support while needing space for parental distress. Joy and apprehension could coexist, with some parents describing relief or a shift from fear for the infant’s health to concerns about independent caregiving. Useful practical instruction did not always translate into feeling prepared, while continuing professional contact could coexist with gaps in coordination between services. Staff, family, and peer support were valued alongside requests for dedicated attention to parental distress. **Conclusions:** Parents’ accounts highlighted the importance of support responsive to individual needs throughout the transition. Priorities for service development include supported participation in care, individualized discharge preparation, coordination across hospital and community services, and access to psychological support.

## 1. Introduction

Preterm birth or the presence of complex clinical conditions represent highly stressful events for parents, associated with the prolonged hospitalization of the newborn in the Neonatal Intensive Care Unit (NICU) [1]. Several studies have documented how the NICU experience is characterized by high levels of anxiety, fear, helplessness, and alteration of the parental role, with a significant impact on the psychological well-being of families [2,3]. However, discharge from the NICU, while representing a long-awaited and hopeful moment, constitutes an extremely delicate transition phase. The transition from the highly technological and supportive environment of intensive care to the home setting exposes parents to new responsibilities and uncertainties, often without adequate structured support [4,5,6]. Qualitative studies conducted in different cultural contexts have highlighted how parents experience persistent feelings of insecurity, loneliness, and unpreparedness in caring for a fragile newborn after discharge [7,8,9,10]. A deeper understanding of parents’ post-discharge lived experiences is therefore essential to inform family-centered transitional care models.

During the NICU stay, parents rely heavily on healthcare professionals, particularly nurses, who play a central role not only in caring for the newborn but also in providing emotional, informational, and educational support preparing them for discharge [11]. The relationship with the care team is described as a source of security and emotional containment, capable of mitigating, at least in part, the traumatic experience of hospitalization [12].

Qualitative studies have already documented parental anxiety, uncertainty, loneliness, and support needs following NICU discharge. However, less attention has been paid to how parents experience the abrupt reduction in the relational, informational, and educational support embedded within NICU care when they assume greater caregiving responsibility at home. Exploring this transition may provide a more nuanced understanding of continuity of care beyond the availability of clinical follow-up alone. This study therefore aimed to explore how parents of preterm or medically complex infants experience the transition from the NICU to home, with particular attention to post-discharge emotional experiences, perceived continuity of care, and support needs.

## 2. Materials and Methods

### 2.1. Design

A qualitative descriptive study was conducted to explore parents’ experiences of the transition from neonatal intensive care to home. The study aimed to understand how parents described returning home, assuming responsibility for their infant’s care, and receiving or seeking support. Accounts of hospitalization were included insofar as they helped contextualize these experiences. The analysis took an experiential orientation: participants’ accounts were approached as descriptions of how they understood and experienced the care pathway.

Braun and Clarke’s thematic analysis method was used [13]. It was chosen to examine shared meanings and differences across parents’ accounts regarding the transition home. Coding was primarily semantic and attentive to meanings explicitly expressed in the interviews, while theme development involved interpreting relationships among these meanings. Theme development was predominantly inductive and grounded in the reading of the interview transcripts. Reporting was guided by the Consolidated Criteria for Reporting Qualitative Research (COREQ) [14], with the completed checklist provided in Table S1.

### 2.2. Study Setting and Recruitment

The study was conducted in a NICU within an Italian tertiary children’s hospital. Care was delivered by a multidisciplinary team, including specialist neonatal nurses, with opportunities for parental involvement in infant care. The institution is not named to limit identification of participating families.

During the recruitment period, A.D. identified potentially eligible parents by reviewing the NICU follow-up programme lists and verified their eligibility against the study inclusion and exclusion criteria. All eligible parents were approached during their infant’s follow-up visit and informed about the study. Those who provided written informed consent were enrolled, and an individual interview was arranged. No further selection was made among eligible parents who consented to participate. The final sample was determined by recruitment within the study period.

Eligibility criteria were being the parent of a preterm or medically complex infant discharged from the NICU within the preceding 12 months, being at least 18 years old, understanding and speaking Italian, and agreeing to an individual interview. Parents whose infant had died during the index hospitalization and those with serious psychiatric conditions considered to interfere with participation were excluded. Both parents of the same infant were eligible and, when both participated, were interviewed individually.

In this study, “medically complex” refers to infants with ongoing medical or surgical conditions at NICU discharge requiring specialized care beyond routine newborn care. Such needs include coordinated specialist follow-up and, where applicable, respiratory support, assisted feeding, or other technology-dependent care. This definition focuses on continuing care needs during the transition from NICU to home, consistent with the neonatal discharge literature [15].

Post-discharge readmissions were not collected systematically. Consequently, this factor could not be used to compare accounts or examine how subsequent events might have influenced recollection.

### 2.3. Data Collection

Individual semi-structured interviews were conducted in person between 15 September and 3 December 2025. Interviews took place in a consultation room located outside the follow-up outpatient clinic, with only the interviewer and the participating parent present. Parents were interviewed separately, including when both parents of the same infant participated, to minimize the potential influence of one parent’s presence on the other’s account. The interviewer, A.D., was a female registered nurse with 15 years of neonatal intensive care experience and postgraduate Master’s-level training in qualitative research. She was not employed in the participating NICU and had no previous clinical, therapeutic, or personal relationship with the participants.

The interview guide was developed from the study objectives, clinical experience within the research team, and consultation with neonatal nursing experts. It addressed the hospitalization and discharge pathway, emotional experiences, support received, unmet needs, and suggestions for improvement. Parents were also asked about their concerns and expectations regarding ongoing care. Because these areas were explicitly included in the guide, their recurrence was interpreted in the context of the questions asked, rather than assumed to indicate entirely spontaneous priorities. The guide is provided in Table S2.

Interviews were undertaken after discharge to allow parents to describe the return home and reflect on the preceding hospitalization from that perspective. This design generated retrospective accounts of the transition; it did not involve repeated prospective observation of changing experiences. Interviews lasted from 10 to 40 min, with a median duration of 20 min [10–30 IQR]. All interviews were conducted in Italian and audio-recorded with consent. Recordings were transcribed in Italian, and identification codes were assigned to the transcripts. No repeat interviews were conducted, no field notes were collected, and transcripts were not returned to participants for comments or corrections. The transcripts were anonymized by assigning identification codes (P01–P38).

### 2.4. Researcher Positioning and Reflexivity

The research team’s clinical background provided familiarity with neonatal care, discharge preparation, and the responsibilities assumed by families at home. The researchers approached the study with an initial expectation that continuity of care would be important during the transition from the NICU to home. This expectation is acknowledged as part of their pre-understanding and as a potential influence on the interpretation of participants’ accounts.

During analysis, this pre-understanding was examined by attending to accounts of support that remained available, successful learning, relief after discharge, and differences between experienced difficulties and anticipated needs. For example, P08 described becoming able to care for the infant independently, whereas P32 described both useful training and insufficient readiness. P03 acknowledged continuing NICU support while criticizing community provision. These comparisons helped limit interpretations of uniformly inadequate preparation or a universal loss of professional support.

No formal reflexive diary was maintained during data collection or analysis.

### 2.5. Data Analysis

The analysis used NVivo 11 to organize the Italian transcripts and coding. All 38 transcripts were independently coded in full by G.G. and C.E.M. The researchers subsequently discussed differences in coding and interpretation and agreed on an initial coding structure, which was reviewed within the research team. Each interview was read as a whole and summarized as an individual case. Numbered responses were used as traceable units for coding, with more than one code assigned when a response contained distinct meanings. Responses were interpreted within the wider interview and in relation to the guide, rather than treated as independent statements. During the coding and theme development process, comparisons were made between researchers and consensus was reached.

Each parent’s interview was analysed as an individual account. For infants whose two parents participated, the accounts were considered in the context of their shared family experience. Within-family similarities and differences were examined alongside comparisons across participants, and differing parental perspectives were retained in theme development.

Relationships between codes were examined within individual interviews and across participants’ accounts. For example, P33 described telephone support and follow-up appointments as reassuring, while also reporting poor coordination of community follow-up. These statements were coded separately as being able to contact staff, receiving follow-up appointments, and encountering gaps in coordination or community care. Considering these meanings together supported a distinction between access to individual professionals and the experience of a coordinated care pathway. Alongside other accounts, including P03’s description of continuing NICU support but insufficient community assistance, this distinction informed the theme “Remaining in contact without always feeling accompanied.”

A further example concerned the relationship between receiving instruction and feeling prepared to provide care independently. P32 valued the practical training received but also described insufficient readiness for discharge. These meanings were coded separately as learning through guidance and feeling insufficiently prepared. Comparison with P08, who described progressing from being afraid to hold the infant to providing care independently, helped retain variation in parents’ experiences. This comparison informed the theme “Being with the infant and learning to provide care” and qualified interpretations of preparation as uniformly inadequate. Accounts that differed from provisional interpretations were retained and used to refine the scope of the themes, rather than being treated as exceptions to be excluded.

Code definitions were also refined where they encompassed different meanings. For instance, requesting a consistent professional throughout the care pathway was distinguished from requesting time and individual attention to ask questions. These distinctions helped preserve differences between parents’ accounts when developing the thematic interpretation.

Coding and theme development used the Italian material. English translations of the selected quotations were prepared for reporting and checked against the original Italian transcripts by E.B. and G.G. to ensure semantic and conceptual equivalence.

A participant-by-theme review was undertaken to document the number of participants contributing to each theme. Each participant was counted once per theme and could contribute to multiple themes. Contributions could include positive experiences, difficulties, contrasting accounts, and suggestions for future care. Ambiguous contributions were excluded from the conservative counts but retained in the qualitative material. These descriptive counts indicate the distribution of clearly attributable contributions across participants.

The analysis produced case summaries, code definitions, a response-level coding matrix, and a record of analytical decisions. The coding tree is provided in Figure S1.

### 2.6. Sample Adequacy and Analytical Transparency

Recruitment was not guided by an assessment of saturation, and no formal information-power assessment was used to determine sample size. The analysis sought an account that reflected both recurring meanings and variation within the available interviews, rather than claiming exhaustive coverage of parental experiences.

Analytical transparency was supported by retaining links between case identifiers, source responses, codes, and thematic interpretations. The absence of repeat interviews, field notes, and participant feedback limited opportunities to clarify ambiguities.

### 2.7. Ethical Considerations

The study was approved by the Local Ethics Committee (protocol no. 21/25, dated 3 September 2025). All participants received detailed information on the objectives and methods of the study and provided written informed consent prior to their participation. Confidentiality was ensured through the anonymization of transcripts and secure storage of audio and text data, accessible only to the researchers involved in the study. This study was conducted in accordance with the ethical principles of the Declaration of Helsinki. Discussing neonatal illness, separation, and the return home could evoke distress, while participants’ continuing reliance on healthcare services could affect how freely they expressed criticism. The interviewer’s lack of a prior care relationship was relevant to this context, although it did not eliminate the possibility of socially desirable responses. The interviewer reported that four mothers cried during their interviews. In each instance, she offered a break, asked whether they needed water or tissues, and checked whether they wished to continue. None expressed a wish to end the interview, and all four interviews were completed.

## 3. Results

Thirty-eight eligible parents were approached for participation; all agreed to participate, provided informed consent, completed an interview, and were included in the final analysis. There were no refusals, withdrawals, or post-enrolment exclusions. The median discharge-to-interview interval was 253 days (IQR 204.25–328.75; range 96–363). The 38 parents represented 34 infants, with both parents participating for four infants. Most participants were female (89.5%, n = 34), with a median age of 35.5 years [29–40 IQR]. Twenty-four (63.2%) were married and 14 (36.8%) were cohabiting. Twenty-two (57.9%) had completed secondary education and 16 (42.1%) held a university degree. Twenty-four (63.2%) were employed. Thirty-one participants (81.6%) were first-time parents.

The infants had a median gestational age at birth of 28 weeks [IQR 25–33] and a median birth weight of 1200 g [IQR 762.5–1915]. Twenty-eight infants (82.4%) were born preterm (<37 weeks’ gestation); fifteen infants (44.1%) had a birth weight below 1000 g. Six infants (17.6%) were from multiple births. The median length of stay was 99 days [IQR 59.5–145.5], with a range of 21–233 days. Clinical conditions were heterogeneous and included complications related to prematurity, gastrointestinal and surgical conditions, congenital anomalies, respiratory morbidity, and neurological conditions. Table 1 details the sample characteristics.

Four themes were developed: (1) Returning home with joy as the focus of fear changes; (2) Being with the infant and learning to provide care; (3) Remaining in contact without always feeling accompanied; and (4) Receiving support while needing space for parental distress. The themes describe different, sometimes coexisting aspects of the transition rather than a sequence experienced identically by all parents. Table 2 summarizes the four themes, the number of participants contributing to each theme, illustrative quotations, and variation across accounts. Using the conservative counting criterion described in the Methods, 38 participants contributed to Theme 1, 36 to Theme 2, 38 to Theme 3, and 35 to Theme 4.

Neither saturation nor information power was formally assessed. The findings therefore represent patterns and variations in the available accounts and do not claim to encompass the full range of experiences following NICU discharge. Accounts included both support that parents valued and needs they considered insufficiently addressed. Requests for future services were distinguished from descriptions of care already received.

### 3.1. Returning Home with Joy as the Focus of Fear Changes

Returning home could restore a sense of closeness and freedom after a period dominated by clinical uncertainty and separation. Parents described happiness at having their infant with them, even when assuming responsibility for care remained frightening. In P04’s account, freedom and fear were part of the same experience:

“*During the hospital stay I felt anxious and afraid. After discharge I felt free because I could finally have my daughter with me, but I was still frightened about having to care for her on my own.*”

(P04)

For some parents, the object of fear changed. Concern about whether the infant would survive gave way to uncertainty about their own ability to provide care. P03 linked this change to happiness about the infant’s progress:

“*During the hospital stay I was afraid that my baby would not survive. After discharge I was happy about the progress he had made, but still frightened that I would not be able to care for him adequately.*”

(P03)

These concerns could relate to ordinary caregiving activities that felt consequential because of the infant’s perceived vulnerability. P05 described being happy to hold the infant again while fearing that she might choke during feeding. Such accounts located uncertainty in the responsibility of caring, rather than demonstrating on their own that no preparation had been provided.

The emotional balance was not uniform. P38 foregrounded relief at leaving hospital:

“*During the hospital stay I was frightened and sad. After discharge I was bursting with happiness; I could finally breathe again.*”

(P38)

P11 distinguished fear from anxiety, describing a reduction in one alongside persistence of the other:

“*During the hospital stay I felt constant fear and anxiety. After discharge the fear disappeared, but the anxiety remained, accompanied, however, by a great deal of joy.*”

(P11)

Thus, the transition was narrated through varying combinations of relief, closeness, and apprehension.

### 3.2. Being with the Infant and Learning to Provide Care

Parents’ accounts connected being physically present with opportunities to experience their parental role and participate in care. Restrictions on contact, visiting time, or space were meaningful because they constrained what parents could do with and for their infant. P04 recognized the clinical demands of intensive care while wanting more time together as a family:

“*More space to be with my little girl. I understand that it is a high-intensity unit and I would never want to put my daughter at risk, but having more moments with her and my husband would have helped me*.”

(P04)

For some, separation was associated with an explicitly described difficulty in feeling a bond. P05 stated:

“*I did not really feel that my daughter was mine. I did not feel the bond that should be there between a mother and her baby, and it was difficult to recover it afterwards; it took a long time.*”

(P05)

Other parents emphasized the wish to spend more time together or participate more actively without describing the bond itself as absent. P02 requested involvement that extended beyond conversation to practical participation during hospitalization.

Learning through guidance could support increasing autonomy. P08 described a clear change in perceived ability:

“*Training in how to care for my baby: I went from not even picking him up to caring for him on my own.*”

(P08)

However, useful instruction did not always translate into feeling sufficiently prepared. P32 valued practical training and emotional support:

“*Practical training in post-surgical care and guidance on feeding and monitoring were fundamental, together with the staff’s emotional support, which helped me manage my anxiety.*”

(P32)

The same participant subsequently requested individualized preparation and noted the following:

“*When we are discharged, I do not think we are ready enough for what awaits us.*”

(P32)

P10 likewise described feeling disoriented at home and explicitly asked for better training before discharge. These accounts identified a gap between receiving help and being ready to apply it independently. Requests included greater involvement, more detailed explanations of everyday care, and preparation adapted to the family. The accounts varied in their description of timing: P01 referred to training throughout hospitalization, while others sought more time or earlier guidance.

### 3.3. Remaining in Contact Without Always Feeling Accompanied

Professional contact could remain available after discharge and provide reassurance in daily care. Telephone advice and follow-up appointments were described as resources for orienting parents when questions arose. As P33 explained:

“*In hospital the staff were attentive and patient, explaining everything step by step. At home I could rely on telephone support and follow-up appointments, which gave me confidence in managing everyday care.*”

(P33)

Nevertheless, access to individual contacts did not necessarily mean that the overall pathway felt coordinated. In the same interview, P33 described difficulties with continuity across community services:

“*I encountered some difficulties with continuity of community care: sometimes the follow-ups were poorly coordinated and clear information was missing.*”

(P33)

P03 similarly distinguished ongoing support from the NICU from the lack of community assistance:

“*Yes, after discharge we were left to ourselves with a child with complex needs. We received no assistance from community services; we only had the NICU supporting us, but that was not enough.*”

(P03)

Other accounts concerned the absence of a consistent person following the pathway:

“*I think the main difficulty was never having a specific ‘key’ person who followed the baby throughout the whole pathway.*”

(P11)

The desired reference was not always located after discharge. P35 described wanting a constant figure within the ward, while P01 wanted opportunities to ask questions calmly with someone’s individual attention.

Information itself could also feel discontinuous. P34 described it as sometimes fragmented or insufficiently timely, without locating all such difficulties in one phase. Taken together, the accounts differentiated being able to contact professionals from receiving coherent guidance and support across the pathway. Suggestions for regular contact or organized follow-up expressed how some parents wished care could be strengthened.

### 3.4. Receiving Support While Needing Space for Parental Distress

Parents drew support from healthcare staff, family members, and others who had experienced neonatal hospitalization. These relationships could provide reassurance and a sense that the experience was shared. As P28 identified:

“*The support I received from my family and from the other mothers who were going through the same situation as me.*”

(P28)

Yet, when asked about difficulties, the same participant named “the lack of psychological support” (P28). P36 similarly described emotional support from staff, family, and other families while reporting the absence of psychological support. Informal and professional relationships were therefore valued without necessarily meeting the need for dedicated attention to parents’ distress.

The experience of staff support was also differentiated. Parents could appreciate care while wanting a more consistent emotional response. P28 requested the following:

“*Greater empathy from healthcare staff during conversations, from everyone, not just some of them.*”

(P28)

Other interviews contained both appreciation and criticism whose circumstances could not be fully clarified from the available responses.

Peer support was sometimes an experience already lived, rather than only a proposal for future care. As P38 explained:

“*The support I received from the other mothers was also fundamental. Although in different ways, they were going through the same situation as me; we gave each other strength.*”

(P38)

The participant also wished for an additional form of support:

“*It would have been useful to have psychological support, if not individually, at least in a group, so that we could support one another.*”

(P38)

Nor was every suggestion a statement of personal unmet need: P37 proposed psychological support for other families without describing a personal lack of it.

Comparison of the accounts of P03 and P28, parents of the same infant, identified shared emotional experiences alongside differences in emphasis. Both described fear during hospitalization and happiness after discharge accompanied by concerns about caring for their infant independently. P03 emphasized gaps in community services despite continued NICU support, whereas P28 foregrounded difficulties in bonding, support from family and other mothers, and unmet psychological support needs. These accounts illustrated complementary concerns within a shared family context, rather than directly opposing assessments of care.

## 4. Discussion

This qualitative descriptive study explored parents’ experiences of the transition from the NICU to home. The four themes describe how joy and apprehension could coexist, how participation in care related to developing confidence, and how valued support could remain available alongside unmet needs. Accounts of successful learning, relief, and continuing professional contact qualified experiences of uncertainty and insufficient support. The findings therefore suggest that parents’ experiences of continuity depend on how available support addresses their needs across the care pathway.

The coexistence of joy and fear is consistent with a meta-ethnography of 12 qualitative studies, which interpreted discharge with a very-low-birthweight infant as a “double-edged sword”, combining anticipation and confidence with uncertainty and challenges at home [16]. Spence et al. likewise described joy alongside anxiety during the first weeks after discharge [17]. In the present study, some parents described a shift from fear for the infant’s survival to concern about their own ability to provide care, whereas others emphasized relief or reduced fear. These variations suggest that the meaning and focus of apprehension deserve attention alongside its persistence. Returning home could restore closeness and a sense of freedom while introducing responsibilities that remained daunting.

The theme concerning presence and learning connected opportunities to be with the infant with participation in care and the developing parental role. Some accounts linked separation with difficulties in establishing a bond; others emphasized wanting more time together and greater practical involvement. Spence et al. similarly described how restricted contact and limited participation shaped parents’ experiences of bonding and preparation [17]. Cleveland’s review identified contact with the infant, inclusion in care, individualized support, and opportunities to practice skills through guided participation as parental needs and supportive nursing practices [18]. The present findings are consistent with this broader understanding of preparation as involving both practical learning and opportunities to become familiar with caring for one’s own infant.

Receiving instruction did not invariably mean feeling prepared for independent care. P08 described increasing autonomy through training, whereas P32 valued practical instruction while also expressing insufficient readiness for discharge. This distinction accords with Aagaard et al.’s finding that parents could continue to experience difficulties at home despite training and information received in hospital [16]. Spence et al. also reported that discharge could feel abrupt and leave insufficient time for preparation [17]. Our accounts varied regarding timing, including training throughout hospitalization and requests for earlier or more individualized guidance. Participants therefore support attention to opportunities to practice and consolidate learning. Feeling unready should prompt exploration of parents’ particular concerns rather than an assumption that no useful education has occurred.

A further distinction concerned access to professionals and the experience of a coordinated care pathway. P33 valued telephone support and follow-up while describing poorly coordinated community care, and P03 acknowledged continuing NICU support alongside insufficient community assistance. These accounts qualify the loss of familiar nursing support described by Spence et al. [17]: professional contact could continue while parents still felt insufficiently accompanied.

Informational continuity also involved having clear, timely explanations and opportunities to ask questions. Requests for a consistent professional across the pathway differed from requests for individual attention during a particular encounter. These findings reinforce Cleveland’s emphasis on information, individualized care, and supportive professional relationships [18]. Furthermore, participants suggest that identifying a contact person and providing information may need to be accompanied by explicit arrangements for communication across services. Because parents’ requests did not consistently specify one profession, these implications concern continuity of care across the multidisciplinary team and community services, including the contribution of nurses.

Helpful relationships did not necessarily meet parents’ needs for dedicated attention to their own distress. Family members, staff, and other parents could provide reassurance while psychological support was still desired. Spence et al. similarly found that the availability of support did not ensure that parents could or wished to use it; privacy, trust, and the timing of an offer influenced their accounts [17]. Our findings suggest a role for opportunities to discuss emotional needs alongside practical care, while recognizing that peer support, empathic communication, and psychological care may address different concerns. Suggestions for psychological services also included recommendations for other families, rather than exclusively personal unmet needs.

These comparisons require attention to context. Aagaard et al. synthesized experiences concerning very-low-birthweight infants across several countries [16], whereas Spence et al. interviewed families of infants born before 29 weeks, 6–8 weeks after discharge, with some experiences shaped by acute COVID-19 restrictions [17]. Our sample included both preterm and medically complex infants, and interviews could occur within 12 months of discharge. These differences limit direct comparison of the prominence or timing of particular experiences. The present study contributes an account of how established transition concerns were expressed within one Italian care setting, particularly how useful support and perceived gaps could coexist.

The findings identify priorities for service development and evaluation: opportunities for supported participation and practice during hospitalization, preparation responsive to individual concerns, clearer coordination between hospital and community services, and accessible attention to parental distress. Parents’ descriptions suggest that reviewing how these elements connect may be as relevant as increasing the number of contacts offered. These are implications drawn from participants’ experiences; the study did not evaluate interventions or establish their effects on parental confidence, mental health, or infant outcomes.

### Strengths and Limitations

The study included parents caring for infants with varied neonatal and clinical conditions and used individual interviews to explore their experiences after discharge.

Several limitations affect the interpretation and transferability of the findings. The study was conducted in a single NICU, and the sample was predominantly composed of mothers. Paternal perspectives were therefore less extensively represented. Clinical heterogeneity broadened the experiences represented but limited detailed exploration of needs specific to particular conditions.

The sample was determined by recruitment during a fixed period; neither saturation nor information power was formally assessed. The absence of repeat interviews, field notes, and participant feedback further limited opportunities for contextualization and clarification.

Interviewing parents after discharge allowed them to reflect on hospitalization from the perspective of caring for their infant at home, but also introduced the possibility of recall bias. The accounts represent retrospective interpretations rather than prospective observation of changes over time.

## 5. Conclusions

Parents’ accounts described the transition from the NICU to home through varying combinations of joy, relief, and apprehension about caring for their infant. Receiving practical instruction did not always translate into feeling prepared, and continued professional contact could coexist with difficulties in obtaining coordinated support. Relationships with staff, relatives, and other parents were valued alongside requests for dedicated attention to parental distress. These findings highlight the importance of considering how available support responds to families’ specific needs throughout the transition.

Within this study context, priorities for service development include opportunities to participate in care and consolidate learning, individualized discharge preparation, clear points of reference across hospital and community services, and access to psychological support when needed.

## Figures and Tables

**Table 1:** Sociodemographic characteristics of participating parents and clinical characteristics of neonates.

Characteristic	n (%) or Median [IQR]
**Parents (n = 38)**	
Age, years	35.5 [29.0–40.0]
*Gender*	
Female	34 (89.5)
Male	4 (10.5)
*Marital status*	
Married	24 (63.2)
Cohabiting	14 (36.8)
*Educational level*	
High school diploma	22 (57.9)
University degree	16 (42.1)
*Employment status*	
Employed	24 (63.2)
Unemployed	10 (26.3)
Parental leave	4 (10.5)
**Neonates (n = 34)**	
Gestational age at birth, weeks	28 [25–33]
Birth weight, g	1200 [762.5–1915.0]
Length of NICU stay, days	99 [59.5–145.5]
*Birth order*	
Firstborn	28 (82.4)
Second-born	5 (14.7)
Third-born	1 (2.9)
*Multiple birth*	
No	28 (82.4)
Yes	6 (17.6)

Legend: NICU LOS–Neonatal Intensive Care Unit Length of Stay.

**Table 2:** Themes, participant contributions, illustrative quotations, and variation in parents’ accounts.

Theme	Participants Contributing (n)	Illustrative Quotation	Variation Within the Theme
T1. Returning home with joy as the focus of fear changes	38	“After discharge I was happy about the progress he had made, but still afraid that I would not be able to care for him adequately.” (P03)	P11 distinguished between fear that had subsided and anxiety that persisted. P38 emphasized happiness and relief, without reporting fear at home.
T2. Being with the infant and learning to provide care	36	“I went from not even holding him to caring for him on my own.” (P08)	P08 described gaining confidence in providing care independently. P32 valued practical training but still felt insufficiently prepared for discharge and wanted more individualized preparation.
T3. Remaining in contact without always feeling accompanied	38	“We received no support from community services; we only had the NICU supporting us, but that was not enough.” (P03)	P03 valued NICU support while describing gaps in community services. P11 reported accessible follow-up and telephone support but wanted a consistent key professional throughout the care pathway.
T4. Receiving support while needing space for parental distress	35	“Psychological support would have been useful, if not individually then at least in a group, so that we could support one another.” (P38)	P28 and P38 valued family or peer support while also expressing a need for psychological support. P37 suggested psychological support for other families without reporting an unmet personal need.

## Data Availability

The data supporting the findings of this study are not publicly available due to privacy and ethical restrictions related to the sensitive nature of the qualitative interview data. Anonymized data may be available from the corresponding author upon reasonable request, subject to applicable ethical and institutional requirements.

## Supplementary Materials

The following supporting information can be downloaded at https://www.mdpi.com/article/10.3390/nursrep16100373/s1 Table S1: COREQ (COnsolidated criteria for REporting Qualitative research) Checklist; Table S2: Interview Guide RSB; Figure S1: Coding tree def.

## Public Involvement Statement

No public involvement in any aspect of this research.

## Guidelines and Standards Statement

This manuscript was drafted against the COnsolidated criteria for REporting Qualitative research) for qualitative research. A complete list of reporting guidelines can be accessed via the equator network: https://www.equator-network.org/.

## Abbreviations

The following abbreviations are used in this manuscript:

NICU	Neonatal Intensive Care Unit
COREQ	COnsolidated criteria for REporting Qualitative research
FCC	Family-Centered Care
IQR	Interquartile range
