# Supplementary material for: Parents’ Experiences After Neonatal Intensive Care Unit Discharge: A Qualitative Descriptive Study
Source: Nurs Rep. 2026 Oct 20;16(10):373. doi: 10.3390/nursrep16100373 (PMC13708508; doi:10.3390/nursrep16100373)
Supplement: Supplementary file 1 [file nursrep-16-00373-s001.zip › Table S2 - Interview guide RSB.pdf]

Table S2 - Interview Guide

**INITIAL AND BACKGROUND QUESTIONS**	
1	Can you briefly describe your experience from recovery in the intensive care unit until discharge?
2	How would you describe the care provided during your hospital stay and in the post-discharge phase?
**EMOTIONAL EXPERIENCES AND PERSONAL EXPERIENCE**	
3	What were the predominant emotions you experienced during hospitalization and immediately after your child's discharge?
4	How did you experience this period, and what were the main emotional difficulties you faced?
**INSTITUTIONAL SUPPORT**	
5	How did the healthcare staff and institutions support you and your family after discharge?
6	Which aspects of the support you received did you find most helpful?
7	Have you encountered any difficulties or critical issues in your relationship with the healthcare system? If so, what were they?
**NEEDS, EXPECTATIONS AND SUGGESTIONS FOR IMPROVEMENT**	
8	Looking at the path followed, what aspects do you think could be improved?
9	Were there any unmet needs or information you wished you had received differently?
10	What suggestions would you give to improve the support offered to families in similar situations?
**CLOSURE AND FINAL REFLECTIONS**	
11	Looking to the future, what are your main concerns or hopes regarding your child’s ongoing care?
12	Is there anything else you would like to add, or feel is important to share regarding your experience?
